# Multi-coil TMS for preclinical applications in ultra-high-field MRI

**DOI:** 10.1162/imag_a_00558

**Published:** 2025-05-02

**Authors:** Victor H. Souza, Heikki Sinisalo, Juuso T. Korhonen, Jaakko Paasonen, Mikko Nyrhinen, Jaakko O. Nieminen, Maria Koponen, Mikko Kettunen, Olli Gröhn, Risto J. Ilmoniemi

**Affiliations:** Department of Neuroscience and Biomedical Engineering, Aalto University School of Science, Espoo, Finland; A. I. Virtanen Institute for Molecular Sciences, University of Eastern Finland, Kuopio, Finland

**Keywords:** automated brain stimulation, magnetic resonance imaging, orientation sensitivity, TMS–fMRI, transcranial magnetic stimulation, rat

## Abstract

Monitoring cortical responses to neuromodulation on preclinical models can elucidate fundamental mechanisms of brain function. Concurrent brain stimulation and imaging is challenging, usually compromising spatiotemporal resolution, accuracy, and versatility. Here, we report on a non-invasive brain stimulation system with electronic control of neuromodulation parameters in a 9.4-T magnetic resonance imaging (MRI) environment. In the imaging scanner, multi-coil transcranial magnetic stimulation (mTMS) is delivered with a 2-coil array, and the MRI signal is measured with a radiofrequency coil. The mTMS can change the stimulus orientation with 1° resolution in a millisecond. Without physically rotating the coils, we evoked orientation-specific muscle responses after cortical stimulation on an anesthetized rat. The mTMS system was successfully implemented and tested with the small-animal MRI, showing minimal interference with B_0_and B_1_^+^fields and uncompromised image quality. A delay of 40 ms between the stimulation pulse and fMRI acquisition—similar or even shorter than those previously described in humans—led to artifact-free images. Concurrent electronically targeted brain stimulation and neuroimaging provides a valuable tool for exploring whole-brain network functions, endorsing more efficient treatment protocols.

## 1 Introduction

Brain processes and diseases engage multiple cortical and subcortical regions that interact in a timely and orchestrated manner, forming complex interconnected networks (Lynn & Bassett, 2019). Instead of focusing on specific areas of the cortex, we need to understand brain function at the network level (Betzel & Bassett, 2017;Gordon et al., 2023;Siddiqi et al., 2022;Yang et al., 2021). Brain functions that span multiple cortical regions are observed across species, which makes preclinical experiments relevant for investigating biomarkers and the effects of treatments in pharmacologically, surgically, and genetically induced disease models (Dawson et al., 2018;Nestler & Hyman, 2010;Surendrakumar et al., 2023;A. Tang et al., 2017;W. Tang et al., 2020). In this context, transcranial magnetic stimulation (TMS) offers a non-invasive way of evoking targeted brain activation, and in combination with functional neuroimaging, it presents a powerful tool for assessing cognition and behavior (Allen et al., 2007;Bergmann et al., 2021;Ilmoniemi et al., 1997;Siddiqi et al., 2021,^2022^;Siebner et al., 2009;Sydnor et al., 2022) and for optimizing therapeutic outcomes (Cash et al., 2021;Siddiqi et al., 2023). While TMS can modulate neuronal activity through processes like long-term potentiation and depression (Funke, 2018;Lefaucheur et al., 2014;A. Tang et al., 2017), neuroimaging methods, such as positron emission tomography (PET) (El Arfani et al., 2017;Krieg et al., 2013;Parthoens et al., 2012;Paus et al., 1997;Tastevin et al., 2020;Wyckhuys et al., 2013), electroencephalography (EEG) (Ilmoniemi et al., 1997;Li et al., 2007;Massimini et al., 2005;Rosanova et al., 2009;Rotenberg et al., 2008), and functional magnetic resonance imaging (MRI) (Bergmann et al., 2016,^2021^;Sydnor et al., 2022), provide spatial and temporal information on the underlying brain activity. EEG has high temporal resolution but offers limited spatial specificity, especially with rodents, and while PET provides higher spatial specificity than EEG, it requires radioactive tracers, hindering large-population studies. In turn, modern ultra-high-field MRI scanners offer superior spatial resolution compared to PET and EEG, enabling non-invasive network-level investigations of healthy and diseased brains (Bergmann et al., 2021;Gordon et al., 2023;Siebner et al., 2009;Tik et al., 2023). Despite its scientific and clinical relevance, to our knowledge, no study has yet implemented interleaved TMS and fMRI in preclinical research.

Combining TMS with fMRI in a preclinical setting poses significant technical challenges (Seewoo et al., 2018). For instance, the restricted space prevents accurately targeting the cortical stimulation inside the scanner bore. It is also extremely difficult to change the targeting parameters during an experiment, and, for instance, studies requiring subsequent pulses on different loci or in different orientations with millisecond-scale intervals are not possible with traditional instruments (Nieminen et al., 2019;Tugin et al., 2021). Moreover, the interaction between the strong static magnetic field and stimulation current in the TMS coil windings leads to high acoustic noise (Nyrhinen et al., 2024) and mechanical stress, causing fractures to the coil structure (seeSupplemental Video), potentially exposing the subject to hazards (Bergmann et al., 2021;Crowther et al., 2013). Notably, these challenges are equally pertinent when combining TMS with fMRI in human studies (Mizutani-Tiebel et al., 2022). The development of multi-locus TMS (mTMS) has enabled electronic control of the induced electric-field orientation and location in the human brain with resolutions better than 1° and 1 mm, respectively (Navarro de Lara et al., 2021;Nieminen, Sinisalo, et al., 2022;Souza et al., 2022). This makes mTMS suitable for automated and highly accurate targeting inside the scanner bore. Recently, a functional MRI coil array has been developed for human mTMS–MRI applications (Navarro de Lara et al., 2023). However, existing mTMS–MRI instrumentation has not been designed with mechanical properties and dimensions applicable to preclinical ultra-high-field MRI settings.

Here, we aimed to (1) develop and characterize an mTMS system with flexible, electronic control of stimulation parameters inside the MRI scanner, seamlessly integrated with imaging sequences; (2) characterize the interplay between the mTMS system and the MRI data acquisition in an*ex vivo*setting; and (3) verify how different TMS pulse orientations evoke distinct muscle responses in an anesthetized rat. This system would enable the placement and use of mTMS in a high-field MRI scanner, offering a unique approach to combine stimulation and imaging in preclinical research with possible translation to human applications.

## 2 Methods

### 2.1 mTMS power electronics and connectivity

The present mTMS power electronics is a two-channel variant of our previously developed six-channel mTMS system for human applications (Nieminen, Sinisalo, et al., 2022). We modified the human mTMS system design to comply with MRI safety and compatibility with minimal ferromagnetic parts from structural components to printed circuit boards (see theSupplemental Materialfor further details). Currently, the system consists of two independent channels (capable of controlling two coils), with an option for adding a third channel. Each channel has an H-bridge module (L. M. Koponen et al., 2017,^2018^) (maximum voltage 1500 V), which drives a current pulse in a single coil in an mTMS coil array. To minimize the electrical interference during mTMS–MRI recordings, the charging of the high-voltage capacitors is interleaved with the MRI acquisitions through a triggering interface.

**Fig. 1.**
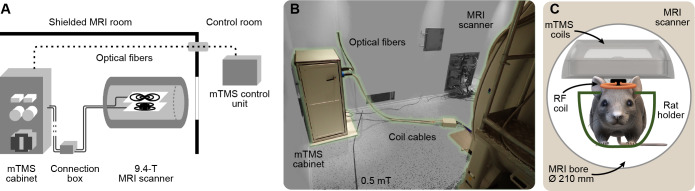
The mTMS–MRI system. (A) Schematic of the mTMS system installation in the MRI shielded room. The optical fibers originating from the cabinet connect to the control unit inside the MRI control room. The mTMS coil array is placed in the scanner bore and connected to the mTMS cabinet via two connection boxes, one inside the bore (not visible in the photo or the schematic). (B) Photo of the mTMS installation inside the shielded room. (C) Illustration of the stimulation and recording coils inside the 210-mm-diameter bore. The transceiver radiofrequency (RF) coil for MRI is attached to the bottom of the mTMS coil case. A custom holder supports the animal and the coils (seeFig. 3A).

A notable change compared to our other mTMS devices is the split installation in the MRI setting (Fig. 1), with the power electronics and the control unit placed in different locations to maintain an environment as free as possible from electromagnetic noise. The power electronics cabinet was fixed in a corner of the shielded MRI room outside the 0.5-mT region, and the control unit was placed inside the operating room. The control unit communicates with the power electronics via fiberoptic links fed through a waveguide into the shielded room. The mTMS cabinet is powered by a standard single-phase mains socket (230 V/50 Hz, 16 A) permanently installed inside the Faraday cage, which has been previously verified to introduce no additional RF noise. The device cabinet was custom-made from aluminum profiles and panels, non-magnetic stainless-steel fasteners (A2 and A4 grades), and polyoxymethylene plastic mounting plates. The electronics were designed to default into a safe state during a sudden absence of power.

To enable fast prototyping of different coil array designs, we designed a connection box inside the MRI bore that allows a simple and safe connection of the coil cables to the cabinet with threaded copper rods and plastic plates for electrical insulation. The mTMS system supports the electronic recognition of different coils via microchips with unique identifiers and connections to digital temperature sensors that can be embedded into the coil array for temperature monitoring.

### 2.2 mTMS coil array

To electronically manipulate the induced electric field (E-field) orientation in the rat’s cortex, we designed and built a 2-coil array. The winding paths for the coils were generated with a minimum-energy optimization method (L. M. Koponen et al., 2018) with wire-density constraints (Rissanen et al., 2023) implemented in MATLAB 2022a (The MathWorks Inc, USA). First, we computed the induced E-field distribution for two coil orientations (0° and 90°) with a small commercial figure-of-eight coil model (MC-B35, MagVenture A/S, Denmark) on a triangular mesh (2568 vertices) located 15 mm from a rat’s cortical surface that was modeled with a 13.7-mm-radius sphere fitted to the local radius of curvature, using a 2562-vertex triangular mesh. For both orientations, we computed the corresponding minimum-energy surface current density in a rectangular plane section (19-cm long and 9.5-cm wide; 1953-vertex triangular mesh) that induces an E-field distribution with focality and intensity similar to those of the commercial coil model. We defined the dimensions of the rectangular plane section to fit inside a small gradient coil set (ø 11–12 cm) typically used in preclinical small-bore MRI systems. For the 0° and 90° coil orientations, we performed the optimization with the rectangular plane located at 15 and 20 mm from the cortical surface, respectively. Then, we decomposed the optimized surface current densities with singular value decomposition and discretized the coil winding paths from each surface current density in 14 isolines. This resulted in two orthogonal figure-of-eight coils (seven turns in each wing).

The 2-coil array has two winding plates (bottom and top) and a case, designed in SolidWorks 2018 (Dassault Systèmes SA, France). The bottom plate thickness is 7.0 mm (including a 2.0-mm-thick bottom), and the top plate is 10.0 mm thick (including a 5.0-mm-thick top). The bottom thickness corresponds to the material thickness below/above the wire grooves. The coil winding paths were milled in polycarbonate plates due to their excellent mechanical properties (75-MPa tensile strength and 2.4-GPa Young’s modulus) necessary to withstand the high stress generated when applying TMS pulses with kiloampere-level currents inside the MRI bore with a 9.4-T static magnetic field. The central region of the plates was reinforced with a 1-mm fluoroelastomer (black surface below the coil wires inFig. 2A) to dampen the impact from the wires. These design choices were based on our finite element model simulations using a physically accurate 3D model of the copper wiring and polycarbonate plates in the coil array by identifying the regions under the highest mechanical stress (M. A. Koponen et al., 2024).

**Fig. 2.**
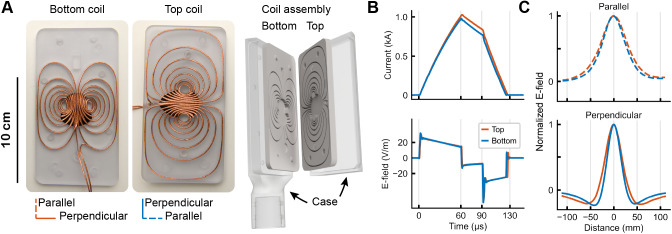
The mTMS coil array, measured current waveforms, and E-field profiles. (A) The two figure-of-eight coils wound with litz wire in the polycarbonate winding plates, and a 3D model of the coil array assembly with the protective case. (B) Trapezoidal monophasic current pulse (upper panel) and its induced E-field waveform (lower panel) for the bottom and top coils inducing an E-field on a 70-mm-radius spherical head model. (C) E-field profiles with directions as indicated in (A) and measured with the TMS characterizer as in (B, bottom). These profiles are used to estimate the induced E-field focality in the direction parallel and perpendicular to the peak E-field.

Each coil was wound with two layers of copper litz wire (1.7-mm diameter; 3-layer Mylar coating; Rudolf Pack GmbH & Co KG, Germany) in the grooves of the coil winding plates (Fig. 2A) and crimped to the connection box placed inside the scanner’s bore. The plates of the coil array were potted with a 2-component silicone-based elastomer (SYLGARD® 184; Dow Inc, USA) so that the 5-mm-thick top of the top winding plate serves as a cover. The coil assembly is housed inside a custom case milled from polycarbonate with a 1-mm air gap to the bottom surface to reduce the mechanical coupling and a 1-mm-thick bottom for additional safety. See theSupplemental Materialfor further details.

After assembling the 2-coil array, we calibrated the electronic control of the induced E-field orientation. Using a TMS characterizer (Nieminen et al., 2015), we measured the E-field distributions at 1000 points in a spherical volume conductor of 70-mm radius, modeling the cortical surface of a human brain. The bottom center of the coil array’s case was 85 mm from the center of the spherical head model. We computed the E-field intensity as its average norm on a trapezoidal monophasic current waveform’s rising phase (60-µs long). For both coils, the monophasic pulse had phases lasting 60 µs (rise) and 30 µs (hold) and a falling phase of 32.5 and 35.2 µs for the bottom and top coils, respectively (Fig. 2B). The difference in the falling phase is mostly due to differing inductances. The focality was computed as the full width at 71% ($1/\text{​}\sqrt{2}$) from the maximum of the perpendicular and parallel E-field profiles (Nieminen et al., 2015) (Fig. 2C).

### 2.3 mTMS–MRI characterization

**Fig. 3.**
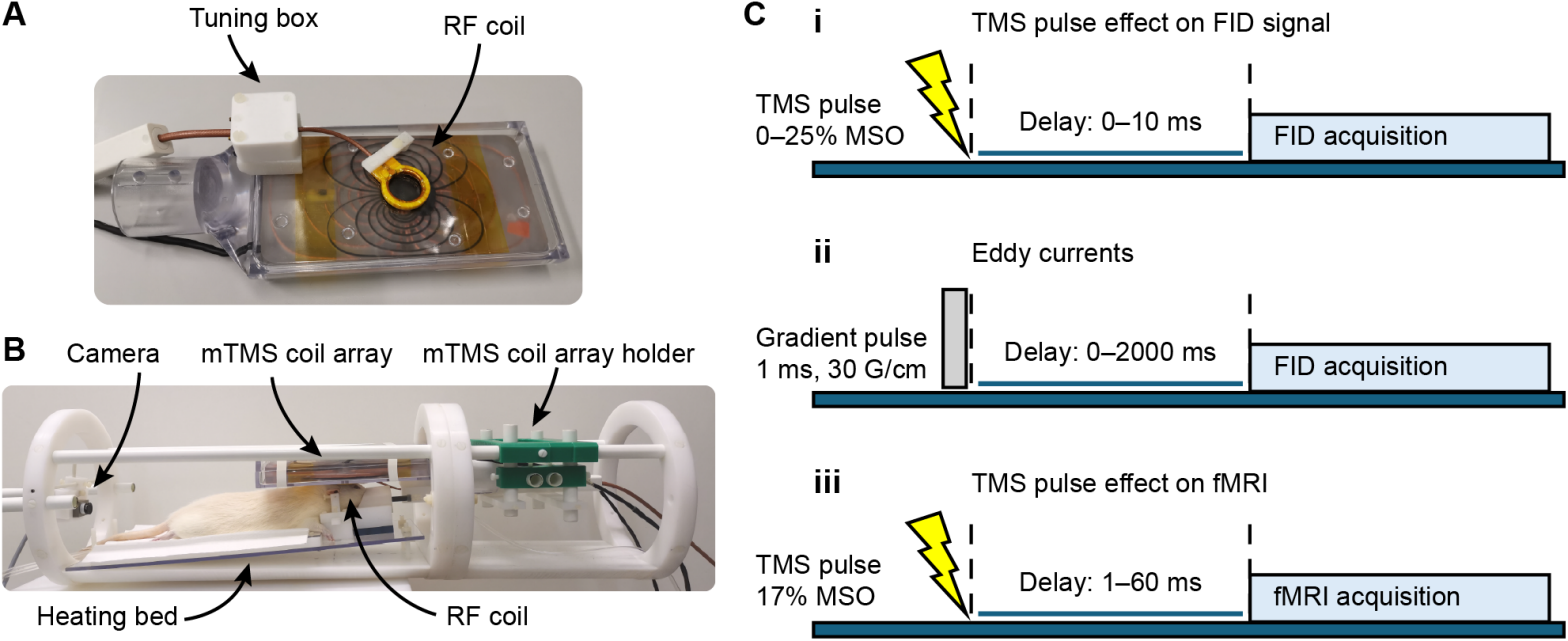
Experimental setup for mTMS–MRI recordings. (A) Custom transceiver radiofrequency (RF) surface coil for recording the MRI signals attached to the center of the mTMS coil array’s case. (B) A custom holder designed to position the stimulation coil array and the MRI RF coil over the rat’s head inside the MRI bore. During the session, an MRI-compatible camera can be used to monitor the anesthetized rat and the coil array. (C) Schematic representation of mTMS–MRI protocols to measure: (*i*) the minimum delay for negligible interference between TMS pulse and FID acquisition, (*ii*) the effect of eddy currents with and without the presence of the mTMS coil array in the scanner bore, and (*iii*) the minimum delay for negligible visually observable deterioration of image quality in EPI fMRI.

To ensure the compatibility of the mTMS device with MRI, we characterized the effect of the mTMS coil array on B_0_and B_1_^+^fields, eddy currents, radiofrequency (RF) noise, and image quality. Additionally, we characterized the required delay to have minimal effect on imaging between the TMS pulse and the RF excitation pulse and between the TMS pulse and fMRI data acquisition. MRI measurements were done with a 9.4-T magnet (bore diameter 31 cm) interfaced with a DirectDRIVE console (Agilent Technologies, Inc, USA) and with either a 12-cm or 21-cm-inner-diameter gradient coil set. A custom-made 400-MHz single-loop transceiver surface coil (Neos Biotec SL, Spain) consisting of a 22-mm inner diameter open loop connected to a separate tuning and matching box with a 6-cm-long coaxial cable was used for RF signal transmission and reception (Fig. 3A). During manufacturing, the tuning and matching range of the RF coil was optimized while it was centered at the bottom of the mTMS coil array case. All imaging was done with an*ex vivo*sample of a rat’s brain embedded in gelatin. A detailed description of the imaging protocols is in theSupplementary Material.

To investigate the influence of the stimulating pulses on the free induction decay (FID) signal, we delivered an mTMS pulse and then acquired the FID signal with varying delays. The RF excitation pulse (1-µs Gauss pulse without slice selection gradient) was given 0–10 ms after the mTMS pulse (Fig. 3C,*i*; 0.5-ms steps, 21 acquisitions with 2.5-s inter-trial intervals). For each mTMS–RF-pulse delay, we tested eight stimulus orientations (−135° to 180° in 45° steps) with five intensities, 0, 5, 8, 17, and 25% of maximum stimulator output (MSO).

To test whether powering the mTMS system or connecting the stimulation coil cables induces RF noise in the MRI signal, an FID was acquired without applying any mTMS pulse (FID_no-pulse_) using the parameters inTable 1with 50 averages. The potential effect of eddy currents originating from the mTMS coil array was assessed by measuring FID after an*x*-,*y*-, or*z*-gradient (1 ms, 30 G/cm) pulse (FID_eddy-currents_) with the parameters inTable 1and delays of 0, 0.25, 0.5, 0.75, 1, 1.5, 2, 3, 5, 10, 20, 50, 100, 500, 1000, or 2000 ms between switching the gradient off and the excitation pulse (Fig. 3C,*ii*), with a 10-s inter-trial interval, and no volume selection. The eddy current measurements were performed with and without the mTMS coil array inside the scanner and are reported as the normalized integral of the power spectrum and phase of the FID signal relative to their corresponding values for the FID signal measured with a delay of 2 s.

**Table 1.** MRI measurement parameters.

Measurement	TR (ms)	TE (ms)	FA (°)	BW (kHz)	FOV (mm ^2 or 3^ )	Matrix size
FID _no-pulse_	500	–	4	250	–	–
FID _eddy-currents_	–	–	10	8	–	–
B _0_ (3D GE)	30	*varying*	20	100	32 × 32 × 32	128 × 32 × 32
B _1_^+^ (GE–EPI)	10	15	*varying*	250	30 × 30	64 × 64
Spin echo	2000	15	90	100	40 × 40	256 × 256
MB-SWIFT	3	~0	5	384	40 × 40 × 40	256 × 256 × 256
GE-EPI	1000	15	60	250	30 × 30	64 × 64

TR: repetition time, TE: echo time, FA: flip angle, BW: bandwidth, FOV: field-of-view, GE: gradient echo, EPI: echo-planar imaging, MB-SWIFT: multi-band sweep imaging with Fourier transformation.

To assess the potential confounding effects of mTMS coil array on the magnetic field homogeneity and RF excitation profile, B_0_and B_1_^+^maps were assessed with and without the mTMS coil array and with imaging parameters inTable 1. B_0_maps were acquired with a 3D gradient-echo (GE) sequence and echo times (TE) of 1.6, 2.1, 2.6, 3.6, 4.6, 6.6, 8.6, and 10.6 ms. B_1_^+^maps were acquired using a GE echo-planar imaging (EPI) sequence with 10 slices of 1-mm thickness and flip angles from 10° to 180° in 10° steps.

High-resolution anatomical images were acquired with a spin-echo and 3D radial zero TE multi-band sweep imaging with Fourier transformation (MB-SWIFT) sequences (Idiyatullin et al., 2015;Lehto et al., 2017) using parameters inTable 1. MB-SWIFT, which is an unconventional MRI sequence, was used because of its virtually zero TE allowed the detection of coil materials and because it is practically free from magnetic susceptibility artifacts (Paasonen et al., 2020). More traditional spin-echo images were acquired with 30 slices of 1-mm thickness. MB-SWIFT had 4000 spokes/spiral, 16 spirals, and 4 RF pulses/spoke. Additionally, GE-EPI sequences commonly used in functional imaging were acquired with parameters listed inTable 1and 10 slices of 1-mm thickness. In a separate measurement, the visually observable effect of mTMS stimuli on GE-EPI fMRI images was assessed by applying mTMS pulses at 45° orientation, an intensity of 17% MSO, and delays of 1, 2, 20, 40, and 60 ms before image acquisition (Fig. 3C,*iii*).

### 2.4 mTMS pulse acoustic noise

We characterized the sound pressure levels (SPL) generated by the mTMS pulses inside and outside the MRI as in (Nyrhinen et al., 2024). We measured the SPL with the open end of a 6.5-m long non-elastic tube in five locations: 4 cm below the coil array’s center and at a 1-m distance (far field), both measurements with the array inside and outside the MRI. The fifth location was with the coil array inside the MRI bore and the tube’s open end in the MRI operation room (seeFig. 8A). In each location, we tested eight different E-field orientations (−135° to 180° in steps of 45°) and six stimulation intensities (13% and 20–100% in steps of 20% of MSO) outside and eight intensities (10–80% in steps of 10% MSO) inside the MRI bore. For high intensities (> 40% MSO), we covered the open end of the tube with a foam earplug to prevent the SPL from saturating the audio interface input. The dampening effect of the foam earplug was quantified in measurements with and without covering the tube’s end, and a correction factor was applied to the SPL measurements with the earplug (see thesection 2.7). To avoid potential damage to the mTMS coil array due to the high Lorentz forces involved, we limited the maximum intensity inside the MRI bore to 80% MSO. We also measured the SPL inside the control room with the mTMS coil array inside the bore using a 20%-MSO stimulation intensity. With the same intensity (20% MSO), we measured the effect of sound-insulating polyurethane foam (HiLo-N40, t.akustik, Germany) inside the scanner and with the measurement tube at a 4-cm distance from the coil array’s bottom. The foam covered the MRI bore walls to dampen the sound wave reflections. See theSupplementary Materialfor additional considerations.

The acoustic measurements were performed with a high-performance microphone (MKE 2 P-C, Sennheiser electronic GmbH & Co KG, Germany) firmly attached to the end of a non-elastic tube (Tress Nobel, 40 bar, length 6.46 m, diameter 6 mm; Tricoflex) and digitized with a high-quality audio interface (RME Babyface Pro, Audio AG, Germany) controlled with custom software written in MATLAB 2022a. To accurately represent the acoustic noise SPL and frequency spectrum, we calibrated our measurement setup to the frequency range of 20–20,000 Hz at the Aalto Acoustics Laboratory (Aalto University, Finland).

### 2.5 Animal experiments

All animal procedures were approved by the Animal Experiment Board in Finland and conducted following the European Commission Directive 2010/63/EU guidelines. Neurophysiological experiments were performed with an adult male Wistar rat (402 g, RccHan®: WIST; Envigo RMS BV, Netherlands). Before the experiment, the rat was anesthetized with isoflurane (5% for induction, 2% for maintenance; Attane Vet 1000 mg/g, Piramal Critical Care BV, The Netherlands) in 30/70 O_2_/N_2_carrier gas. Subsequently, anesthesia was switched to urethane (1250 mg/kg i.p.; Sigma-Aldrich, Corp, USA). Sufficient depth of anesthesia was periodically confirmed by pinching the hind paw to observe the loss of limb contraction reflex. The rat was placed on a warm water circulation pad (37°C; Corio CD, Germany) to maintain a normal body temperature.

### 2.6 Electrophysiological recordings

The head of the rat was fixed to our custom-made holder with ear and bite bars. The mTMS experiment started 30 min after the initial isoflurane anesthesia induction. EMG was recorded with disposable monopolar needle electrodes (SDN electrodes, stainless steel; Inomed GmbH, Germany) inserted into the belly of the biceps brachii muscle in the depilated forelimbs’ left and right paws (Boonzaier et al., 2020;Rotenberg et al., 2010), as shown inFigure 9C. The muscle belly was determined by palpation of the extended forelimb. The references were placed between each paw’s second and third digits, and the ground was inserted in the tail’s base. EMG signals were digitized with a NeurOne Tesla (Bittium Biosignals Ltd, Finland) with a 10-kHz sampling frequency and 2.5-kHz low-pass filtering.

We measured the effect of mTMS stimulus orientation on the amplitude and latency of motor-evoked potentials (MEPs) in the anesthetized rat outside the MRI bore. We first mapped the scalp location on the right hemisphere, showing the highest MEP amplitudes with the center of the coil array at about 5 mm lateral to the bregma and with stimulation intensity set to 67% MSO. Stimulation intensity and the coil array’s location were adjusted until we detected MEPs on the left biceps brachii muscle with visible limb twitches. With the coil array location fixed on the right hemisphere’s hotspot, we searched for the resting motor threshold (RMT) as the minimum stimulation intensity evoking at least three out of six MEPs with amplitude over 10 µV. We mapped the hotspot on the left hemisphere following the same process as above but with stimulation intensity set at 110% RMT of the right hemisphere. The interval between consecutive pulses was 4.0–5.0 s. Finally, we applied five single pulses with 110% of the right hemisphere’s RMT in each of eight stimulus orientations (−135° to 180° in steps of 45°) over the hotspot at the left and right brain hemispheres while recording electromyography (EMG) from left and right biceps brachii muscles.

### 2.7 Data analysis

#### 2.7.1 MRI data

To assess mTMS interference on FID signals, Fourier transforms were applied, and power spectra integrals were calculated and normalized to spectra without mTMS pulses. Power spectra acquired after an mTMS pulse were considered corrupted if their ratio to the power spectrum without a preceding mTMS pulse was below 95%. The B_0_maps were computed from the development of signal phase as a function of TE and presented as the shift in the Larmor frequency (γΔB_0_), where γ is the proton’s gyromagnetic ratio (42.58 MHz/T) and B_0_is the static magnetic field strength (9.4 T). The B_0_homogeneity was quantified as the standard deviation (σ) of γΔB_0_in pixels inside an ellipsoid region of interest (ROI) encompassing the rat’s brain in the phantom, seeFigure 6A. The B_1_^+^maps were computed from the signal development as a function of nominal flip angle and presented as the ratio between the nominal and actual flip angles. MB-SWIFT images were reconstructed as previously described inPaasonen et al. (2020).

#### 2.7.2 Acoustic data

The SPLs were calculated without weighting, that is, Z-weighting (Acoustical Society of America, 2014), and as the average of the maximum SPLs across three separate mTMS pulses. The frequency spectrum was measured with a time window spanning 3 ms before and 40 ms after the pulse. The window length was approximately the time for the sound waves to travel twice the length of the measurement tube. Data samples of equal length were selected to calculate the frequency responses of the background noise. The samples were cosine-filtered using the Tukeywin function to reduce artifacts from cutting the signal. Then, the filtered signal was used to create the 1/3 octave spectra. SPLs from all stimulus orientations using 300 V stimulation intensity were compared with measurements done with the same intensity and orientations using an ear plug as a dampener to get a correction factor for the effects of the earplug. The correction factor (48 dB(Z)) was then added to SPLs measured using the earplug. Measurements with the earplug were left out from the 1/3 octave spectra because the earplug altered the frequency responses.

#### 2.7.3 MEP data

The MEP data were pre-processed by removing the pulse artifact from the continuous EMG signal using the TMS–EEG Artefact Removal tool (Version 1.0; Mega Elektroniikka Oy, Finland). The exponential decay model coefficients were set to 0 and band-pass filtered (3rd-order Butterworth filter, 15- and 1000-Hz cut-off frequencies). The EMG signal was divided into epochs from 5 ms before to 20 ms after the TMS pulse. MEPs were visually inspected, and epochs showing large artifacts, visible heartbeats, or having MEPs with peak-to-peak amplitude smaller than 10 µV were discarded from further analysis. In total, 33 MEPs were discarded from the 80 recorded. We extracted the peak-to-peak amplitude from the remaining MEPs and manually annotated the onset latency.

All data were processed and analyzed with custom MATLAB scripts.

## 3 Results

### 3.1 MRI-compatible mTMS system

The resulting E-field spatial distributions in the 70-mm-radius sphere surface used for human brain modeling for the orientations 0° (bottom coil), 45° (both coils), and 90° (top coil) are shown inFigure 4A. Computed on the 13.7-mm-radius spherical surface model of the rat brain, the bottom and top coil focalities in the direction perpendicular to the peak E-field were 10.8 and 12 mm, respectively; in the parallel direction, they were 23.7 and 28 mm, respectively. The top coil (16.7 µH, 133 mΩ) required approximately 37% more voltage than the bottom coil (12.7 µH, 122 mΩ) to induce the same E-field measured with the TMS characterizer.

**Fig. 4.**
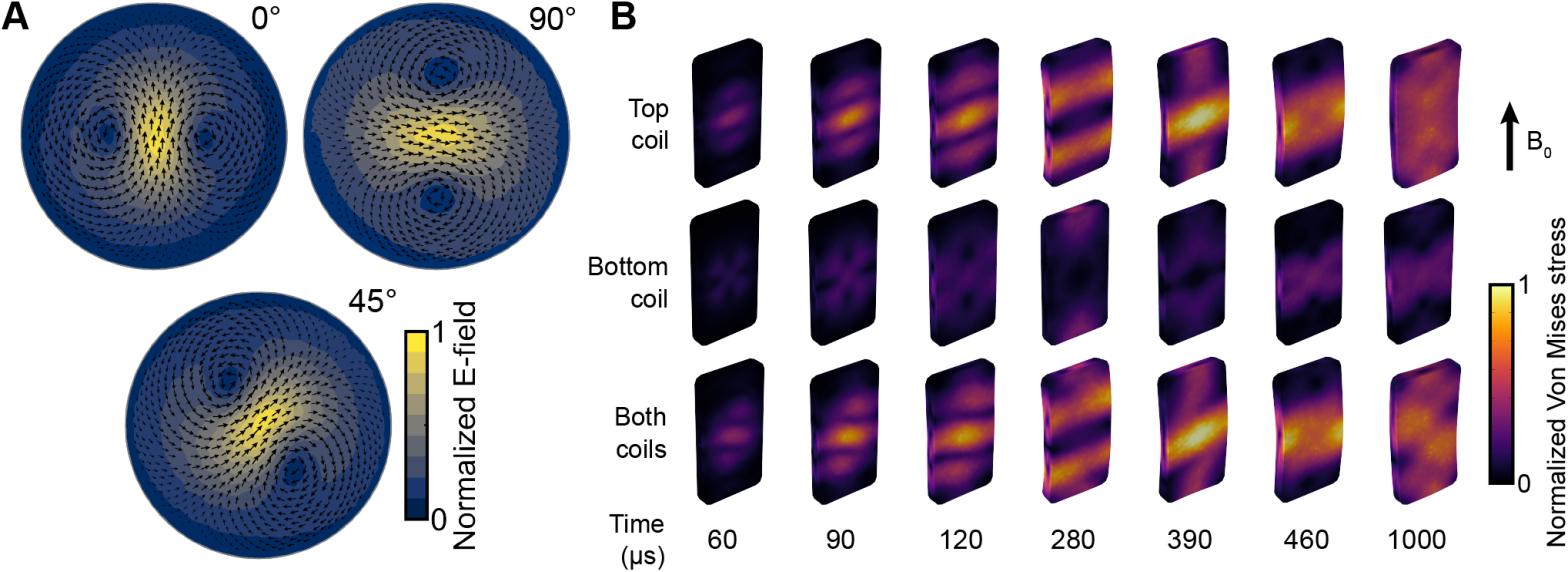
The measured E-fields and simulated stress on the coil plates. (A) E-field spatial distribution measured with the TMS characterizer on a 70-mm radius spherical head model. The 0°, 90° and 45° correspond to the E-field orientation induced with the bottom coil, top coil, and both coils together, respectively. (B) Simulated stress distributions on the coil winding plates inside the MRI bore with a 9.4-T static magnetic field (B_0_ ) when mTMS pulses were applied to each coil separately or to both coils simultaneously (modified fromM. A. Koponen et al. (2024)). The time is relative to the mTMS pulse onset as in the current waveform illustrated inFigure 2B.

As described byM. A. Koponen et al. (2024), the top coil or with both coils fired together results in higher mechanical stress on the plates than for the bottom coil alone. The peak stress values with a 100% MSO monophasic stimulation pulse (rise time: 60 µs, hold: 30 µs, fall: 32.5 for the bottom and 35.2 µs for the top coil; seeFig. 2C) were 32 MPa, 71 MPa, and 71 MPa for the bottom coil, top coil, and both coils together, respectively. The change in stress distribution over time suggests that the coil plates vibrate, deform (Fig. 4B), and may eventually break.

### 3.2 Interplay between mTMS and MRI

**Fig. 5.**
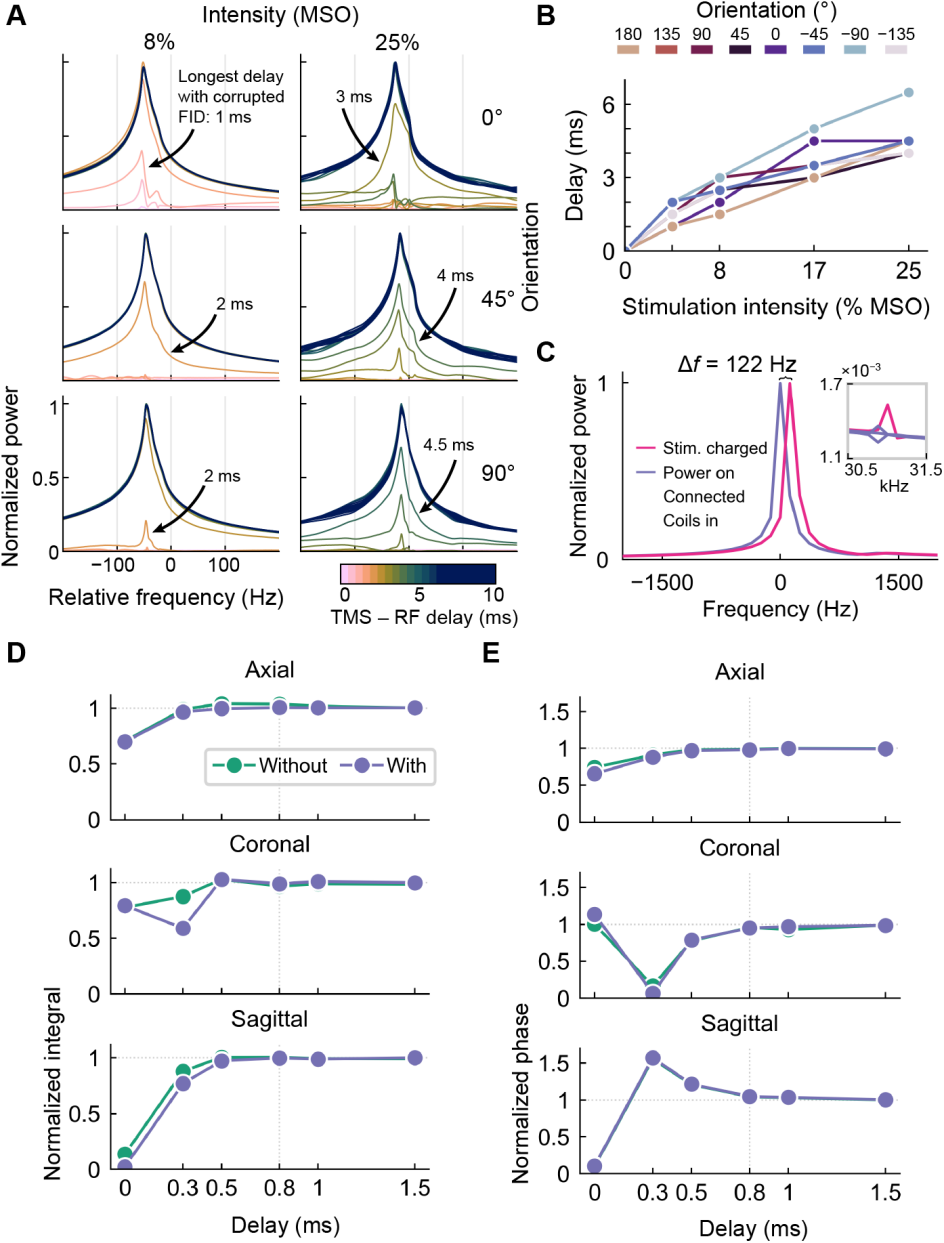
Effect of mTMS pulse and equipment on the MRI signal and performance. (A) Spectra recorded with multiple mTMS pulse intensities, orientations, and delays between the mTMS and RF pulses. The numbers above the arrows indicate the longest delay (in milliseconds), resulting in a corrupted power spectrum. Power spectra recorded after an mTMS pulse were classified as corrupted if the ratio of their integrals, compared to that of the power spectrum obtained without a preceding mTMS pulse, was less than 95%. At 0° orientation and with a stimulation intensity of 25% MSO. (B) Minimum delay between the mTMS pulse and the RF excitation pulse to obtain an artifact-free FID signal at different E-field orientations. Stimulation intensities are measured as % of the maximum stimulator output (MSO). (C) MRI signal normalized power spectra recorded with the mTMS coils inside the bore and under the following conditions: coils disconnected from the power cabinet (green line), mTMS coils connected to the power cabinet (orange), coils connected and mTMS system powered on (purple), and finally with the capacitors charged (pink). All power spectra are approximately the same, except when the stimulation capacitors are charged, the peak frequency shifts by 122 Hz, and a small peak appears at about 31 kHz (zoomed chart). The effect of eddy currents was measured as (D) the normalized integral of the power spectrum and (E) the normalized phase of the FID signal at specific delays between switching the magnetic field gradient (axial, sagittal, or coronal) off and the RF excitation pulse, without (solid green line) and with the mTMS coil array (dashed purple line). The green line and markers (data without the mTMS coil array) may be hidden under the purple ones due to overlapping data, as in the sagittal panel. The integral and phase were normalized relative to their corresponding values measured with a delay of 2 s.

The close vicinity of the coil array shifted the RF coil’s resonance frequency by 7 MHz, which was considered while optimizing the RF coil tuning and matching range during the manufacturing phase. The FID power spectrum was distorted in most cases up to 4.5 ms after applying mTMS pulses at 25% MSO (Fig. 5A). Stronger intensities required longer delays, which also depended on the induced E-field orientation. A TMS pulse with the top coil alone (−90° orientation) corrupted the FID power spectrum up to 6 ms (25% MSO;Fig. 5B). The corrupted power spectra for this pulse are not shown inFigure 5Abecause the 6-ms trace is just slightly corrupted and cannot be visually distinguished from the longer delays. A pulse with two coils simultaneously, for example, in 45° orientation, corrupted the power spectrum at delays up to 2 ms (8% MSO;Fig. 5A–B).

A 122-Hz shift in the resonance frequency and additional small peaks around ± 31 kHz were observed in the FID signal when the mTMS capacitors were charged and the stimulation coils were located in the scanner bore (Fig. 5C), indicating potential confounding effects on imaging. The effects on the FID signal from non-charged coils in the scanner bore appeared negligible. The potential eddy currents originating from the mTMS coil array had practically negligible effects on magnetic field gradient performance in all three directions. In sagittal and coronal orientations, the phase and integral of the power spectrum were the same with and without the mTMS coil array inside the bore for delays of at least 0.5 ms between the gradient pulse and the FID acquisition. In axial orientation, the integral of the power spectrum was slightly distorted by eddy currents up to 0.8 ms, while the phase was unaffected with a delay of at least 0.3 ms (Fig. 5D–E).

**Fig. 6.**
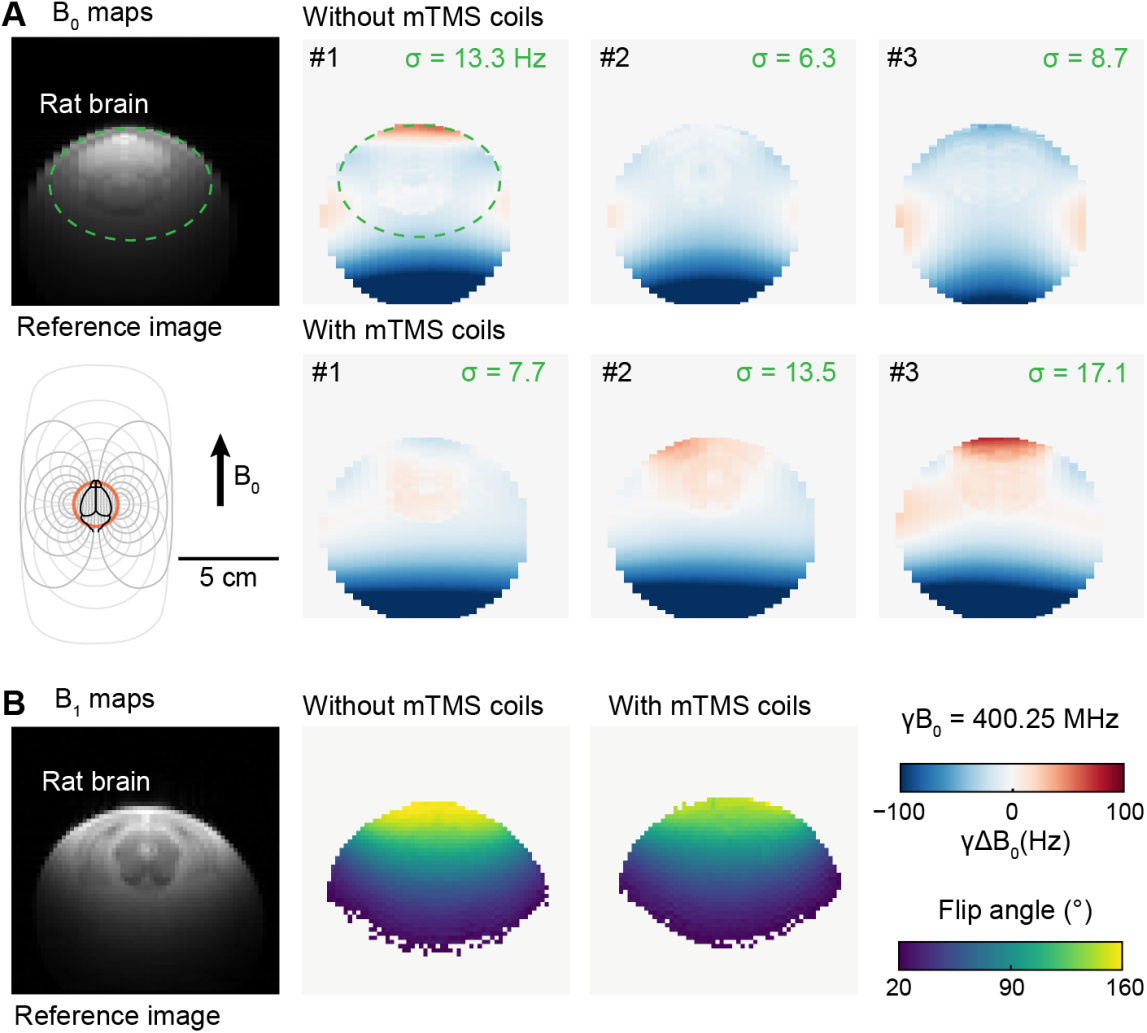
Effect of mTMS coils on the homogeneity of B_0_and B_1_^+^with and without the mTMS in the MRI bore. (A) A reference anatomical MRI indicates the area with the rat brain and the dashed green circle shows the region of interest (ROI) where the standard deviation (σ) of the measured B_0_homogeneity was computed. Below is a schematic representation of the mTMS coil array (windings as gray lines) and RF coil (orange circle) positioning relative to the rat brain phantom. The B_0_homogeneity maps are shown for three coronal slices as the deviation from the Larmor frequency (γΔB_0_ ). An extended set of B_0_maps in axial, coronal, and sagittal orientations are depicted in Supplementary Material (Figs. S1–S9). (B) A reference anatomical MRI followed by two representative coronal slices of the B_1_^+^maps. The attenuation of transmission power, that is, decrease in flip angle, toward the lower parts of the sample is characteristic when a single-loop surface transmit-receive coil is used above the sample. Additional slices are depicted in Supplementary Material (Figs. S10–S12).

**Fig. 7.**
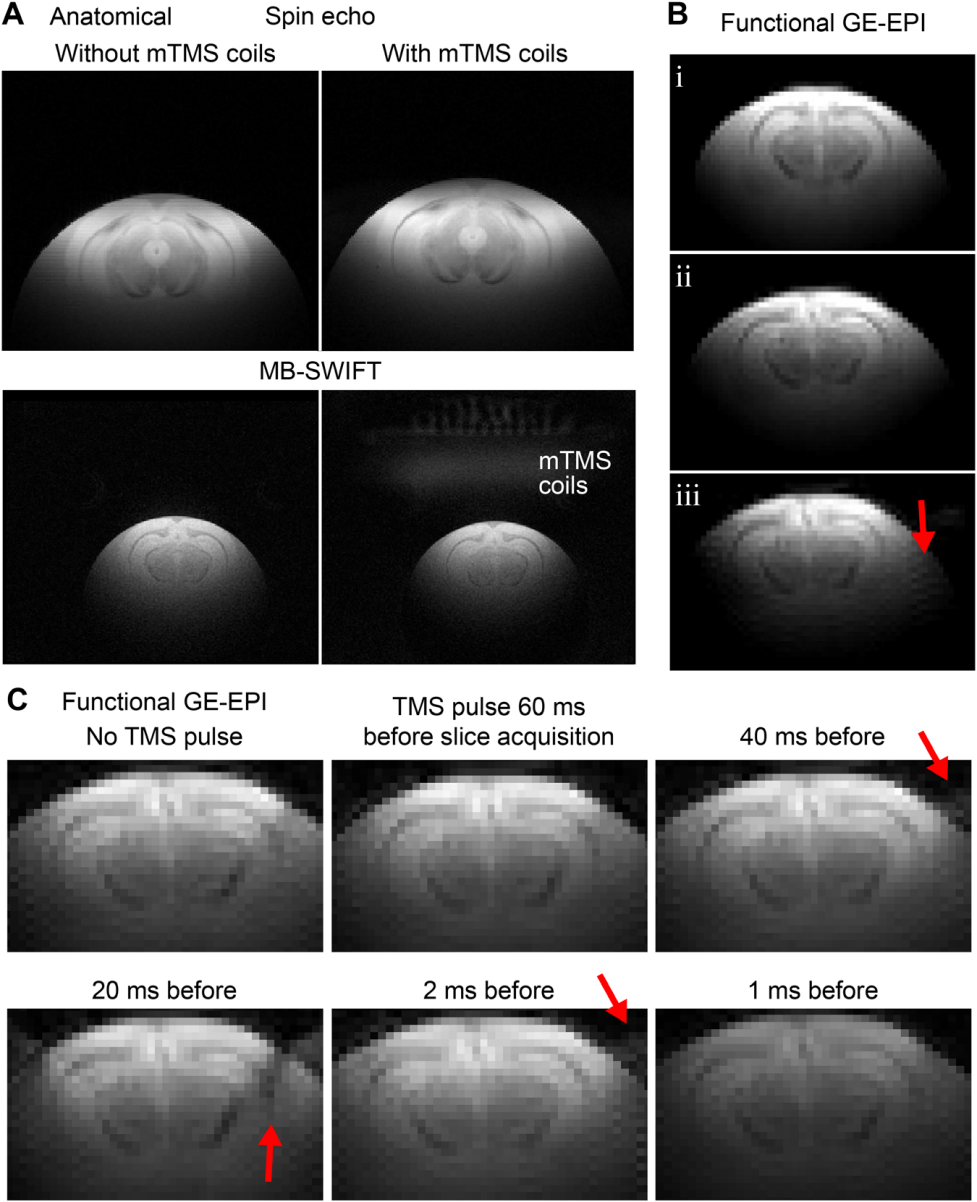
Anatomical and functional images and corresponding mTMS-induced artifacts. (A) Spin echo and MB-SWIFT images with and without the mTMS coils inside the bore. (B) Functional gradient echo (GE) echo-planar imaging (EPI) without the mTMS coils (*i*), with the mTMS coils connected to the powered-on electronics (*ii*), and same as in (*ii*) but with capacitors charged (red arrow point to stripe artifacts). (C) Functional GE-EPI without and after mTMS stimuli at different delays. The red arrows point to visible imaging artifacts resulting from the mTMS pulse interference with short delays.

The B_0_maps indicate only a minimal increase in the standard deviation of the field strength in the presence of the coil array (Fig. 6A), suggesting that the presence of mTMS coil does not prevent sufficient homogenization of the B_0_field for EPI fMRI. The B_1_^+^maps suggest a similar distribution of the transmission power, that is, flip angles, across the sample with and without the mTMS coil array in the bore (Fig. 6B), showing only the expected minor decrease in FA with the mTMS coil array in the bore (Navarro de Lara et al., 2020). There was no abnormal increase or loss of RF focusing in both conditions. In line with these observations, the high-resolution anatomical images confirmed that no compromise in image quality can be visually observed in spin echo or MB-SWIFT images (Fig. 7A). The mTMS coils can be seen with MB-SWIFT sequence (Fig. 7A. bottom right), as the zero TE sequence is sensitive to protons with fast relaxation rates.

In agreement with the results shown inFigures 5–6, we did not detect critical image artifacts in functional images acquired with GE-EPI while the mTMS coils were connected and its electronics were powered on (Fig. 7B,*i*–*ii*). However, while capacitors were charged, a subtle stripe pattern appeared in the EPI image (Fig. 7B,*iii*), and the sample shifted roughly one voxel in the image in the phase-encoding direction. The stripe pattern possibly originates from leaking currents from the capacitors, inducing the additional peaks at ±31 kHz (Fig. 5C). The spatial shift of the sample likely originates from the 122-Hz shift in the resonance frequency (Fig. 5C), which mostly affects the low-bandwidth phase-encoding direction in EPI sequences. To avoid the stripe pattern and spatial shifts, the mTMS capacitors can be charged just before the stimulation is applied, when there is no MRI acquisition, and kept discharged otherwise. Moreover, we found that an mTMS pulse affects the GE-EPI acquisition if done with an interval shorter than 40 ms (Fig. 7C); however, being still suitable for most preclinical fMRI experiments.

### 3.3 Acoustic noise by the mTMS pulse

**Fig. 8.**
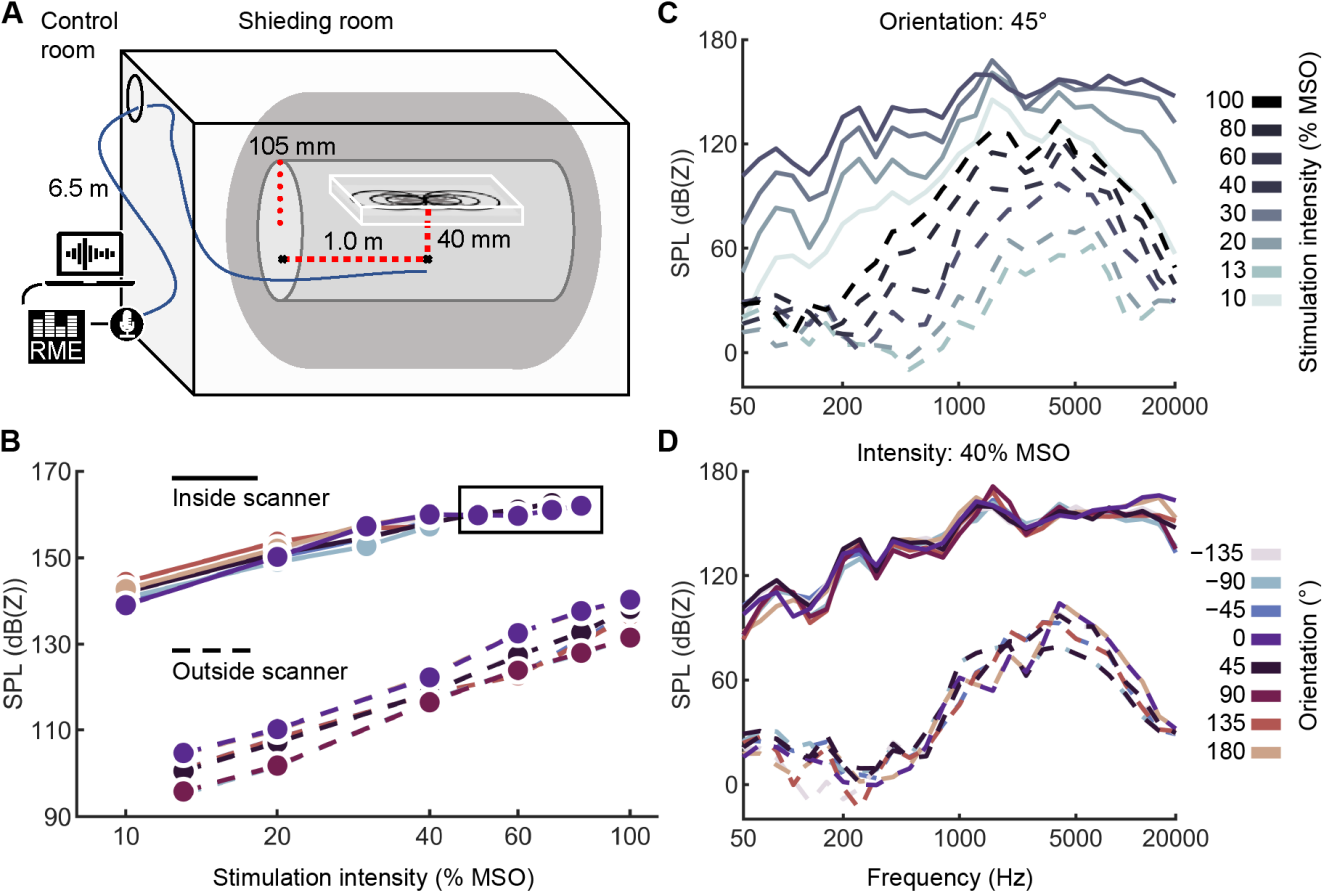
Acoustic noise from the mTMS pulses. (A) The acoustic noise measurement setup inside the MRI bore with the open end of the measurement tube pointing towards the coil array and placed inside the MRI bore in the two positions indicated by the black dots connected with red dashed lines for 1) near-field and 2) far-field measurements. The tube orientation and position in the image are for illustration purposes only. (B) Peak SPLs measured outside and inside the MRI scanner with the open end of the tube at 4 cm below the coil array with different stimulation intensities and orientations. The measurement points inside the square were performed using an earplug at the open end of the tube and corrected (48 dB(Z)) for the corresponding dampening in SPL. (C) For different intensities, 1/3 octave spectra of the acoustic noise from mTMS pulses at 45° orientation (bottom and top coils together). (D) 1/3 octave spectra for mTMS pulses at 40% MSO with varying stimulus orientations. In all charts, dashed and solid lines indicate measurements outside and inside the MRI scanner, respectively.

The peak SPL measured outside the MRI bore (without the B_0_field) was 142 dB(Z) at a 180° stimulus orientation and with 100% MSO stimulation intensity (Fig. 8B). Inside the bore, the highest peak SPL was 167 dB(Z), measured with a 0° pulse orientation (earplug as a dampener) using 80% MSO stimulus intensity. The presence of the static magnetic field increased the SPLs by 44 dB(Z) on average, with stimulation intensities of 20% and 40% MSO (Fig. 8C). The effect of orientation across all stimulation intensities was 9 dB(Z) on average, 0° being the loudest and −90° the quietest (Fig. 8D). In the control room, SPLs were below 85 dB(Z), and it was not possible to distinguish them from the background noise with our measurement system. Thus, the noise is reduced by at least 60 dB from the scanner center to the control room. The sound-insulating foam lowered the peak SPL measured below the coil by an average of 4 dB(Z). At a 1-m distance, the peak SPLs inside and outside the bore were attenuated by 20 dB(Z) and 23 dB(Z) on average, respectively.

### 3.4 Effects of stimulus orientation on motor response

**Fig. 9.**
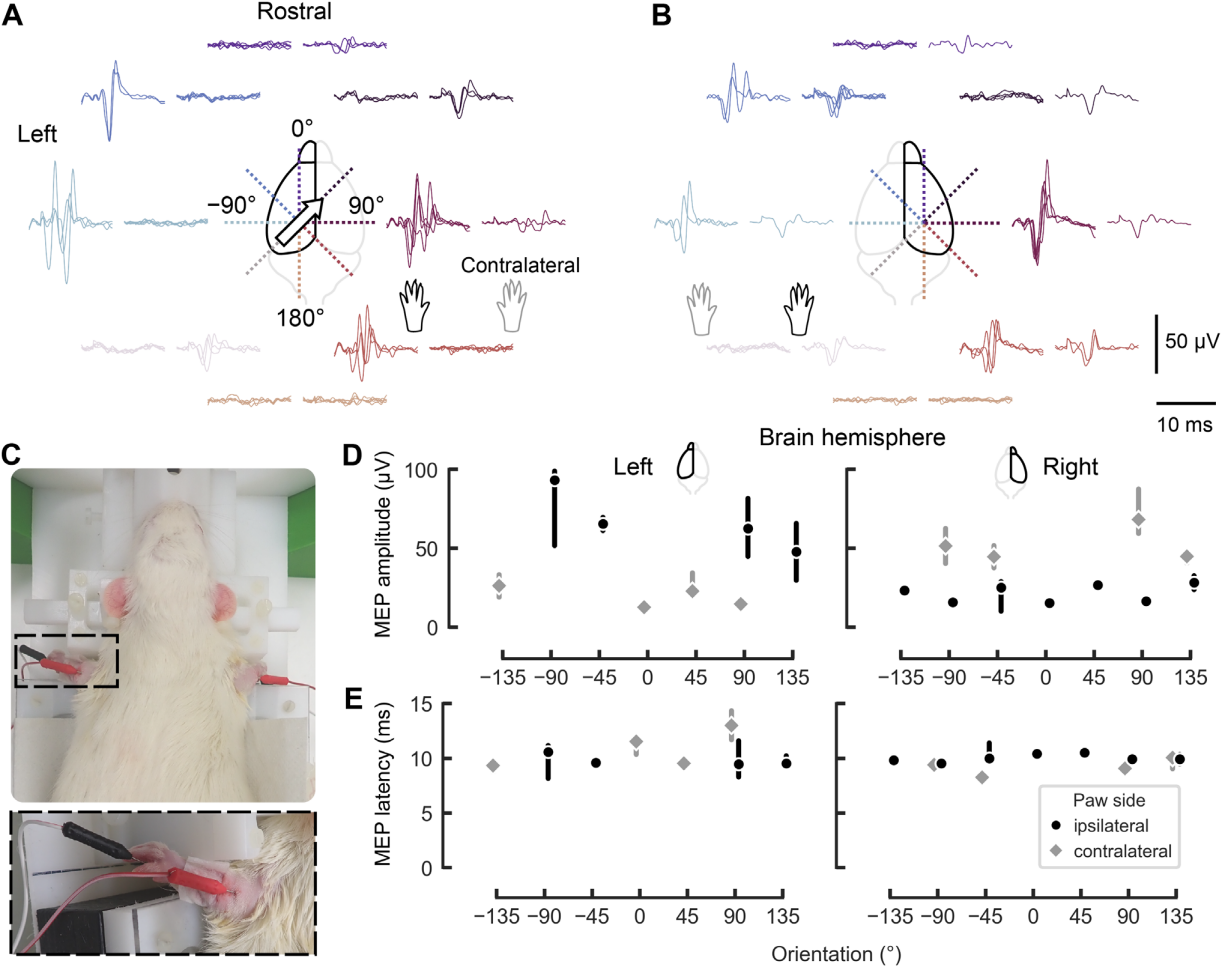
Effect of mTMS pulse orientation on MEPs. MEPs recorded from the rat’s left and right biceps brachii with mTMS pulses on the (A) left and (B) right rat brain hemispheres. (C) Photos depicting the rat placement on the holder and the needle electrode placement on the right and left front paws. The dashed square highlights the electrode placement. (D) MEP amplitude from ipsilateral (black circle) and contralateral (gray diamond) paws after stimulation of the rat’s left and right brain hemispheres with stimulus orientations from −135° to 180°. Markers represent the medians across trials, and the error bars represent the 95% confidence interval. (E) Latency for the corresponding MEP amplitudes. Missing latency values result from MEPs with zero amplitude or trials rejected during signal preprocessing. The biggest contralateral MEPs were recorded with mTMS pulses on the right brain hemisphere (B) and with the E-field orientations 45°, 90°, 135°, and −90°.

We observed that the MEP amplitude considerably depended on the stimulated brain hemisphere and the E-field orientation (Fig. 9A–B). On the right brain hemisphere, contralateral MEPs with the highest amplitudes were evoked at −90°, −45°, 90°, and 135°, while no MEPs were detected at the remaining orientations. In turn, the left hemisphere showed the highest contralateral MEP amplitudes for mTMS pulses at 45°, 90°, and −135°, with no MEPs at −90°, −45°, and 180° (Fig. 9D). We detected ipsi- and contralateral MEPs in both hemispheres with latencies of about 10 ms (Fig. 9E).

## 4 Discussion

We introduce and demonstrate the first in-situ mTMS–MRI setup for concurrent stimulation and imaging of small animals in an ultra-high-field MRI scanner (9.4-T static field). The electronic control of TMS stimulus parameters with the coil array inside the MRI bore solves critical limitations of concurrent TMS–MRI applications. For instance, with specialized multi-coil arrays (Nieminen et al., 2019;Rissanen et al., 2023), distinct cortical areas can be subsequently targeted within milliseconds with interleaved imaging sequences, allowing causal assessment of network brain functions (Siddiqi et al., 2022). With electronic targeting, we also minimize the need to reposition the stimulating coil physically. Such repositioning requires the operator to remove the experimental setup from the bore, which is time-consuming and prone to errors.

The mTMS–MRI installation with the cabinet inside the shielded room and the control unit outside provides significant advantages compared to existing systems (Bestmann et al., 2003;Navarro de Lara et al., 2017,^2023^). This installation minimizes the length of the coil cabling, whereas commercial MRI-compatible TMS systems typically utilize extra-long coil cables and introduce the coil into the MRI room via a waveguide combined with a low-pass filter. This has been reported to result in a loss of up to 15% in stimulation strength (Bergmann et al., 2021). The stimulation intensity is a critical limiting factor in TMS in preclinical applications due to the reduced electromagnetic coupling between the animal’s brain and the stimulation coils. Thus, even minor improvements in stimulation intensity might enable a broader range of stimulation protocols. Another crucial improvement is that the computer that controls the mTMS device from the MRI control room allows flexible programming of mTMS pulse waveforms and sequence protocols. This feature eliminates the need for the operator to frequently enter and exit the shielded room during experimental sessions, reducing exposure to potential health risks, such as electric hazards and high acoustic noise levels (Nyrhinen et al., 2024). We have not identified any RF noise in MRI recordings after installing the mTMS power electronics inside the scanner room. The cabinet is located outside the 5-G line and when it is not in use, it is disconnected from mains electricity. Thus, the presence of the cabinet in the room is unlikely to generate detectable noise in the RF power spectrum.

A unique characteristic of our system is the electronic control of the E-field orientation in the rat cortex enabled by the 2-coil array inside the MRI bore. We combined rigid polycarbonate plates with a layer of fluoroelastomer and potting with a special silicone for improved impact absorption. This layered design withstood stimulation intensities up to 80% of MSO in all orientations. This is a significant improvement compared to our first prototypes manufactured with only polyoxymethylene (POM) and potted with epoxy, which fractured at about 40% MSO (unpublished data). Despite the apparent large size of the 2-coil windings, their induced E-fields with 10-mm focal width computed on a 13.7-mm rat brain’s spherical model are similar (Boonzaier et al., 2020;L. M. Koponen et al., 2020) or narrower (Meng et al., 2018) than other custom coils tailored for rodent TMS. With a similar focality as existing coils, we demonstrated the feasibility of electronic control of the stimulus orientation to generate MEPs at specific E-field orientations (seeFig. 9A–B). Even so, these relatively broad E-fields might not allow selective activation of spatially close cortical network nodes in the small rodent brain unless the cortical neuronal populations in question have an orientation specificity as demonstrated in humans (Cerins et al., 2024;Pieramico et al., 2023;Souza et al., 2022;Weise et al., 2023). The relatively broad E-fields may also elicit widespread blood-oxygen-level dependent responses that are not specifically linked to the target area. To address these issues, we anticipate that future efforts will significantly improve the mTMS focality through miniaturized coil designs, which is technically challenging considering the high current requirements (Wilson et al., 2018). We should note that our first effort was to enable*safe and suprathreshold*stimulation, considering the strict requirements for MRI compatibility and to withstand the excessive Lorentz forces inside the bore. In applications not requiring high focality, it might be best to trade the focality fully for efficiency (Nurmi et al., 2021), as such coils can evoke reliable MEPs in rats with relaxed durability requirements (Nieminen, Pospelov, et al., 2022). Importantly, the multi-channel TMS system is flexible and can be utilized with multiple coil array combinations designed for specific purposes, such as precise electronic cortical mapping (Nieminen, Sinisalo, et al., 2022;Rissanen et al., 2023).

Our*ex vivo*characterization indicates that the MRI-compatible mTMS system suits conventional preclinical MRI studies with commonly used structural and functional sequences. We observed minimal to no differences across the B_0_and B_1_^+^fields or the eddy currents with or without the mTMS coil array in the bore, resulting in no visually discernible differences in the image quality. Although the MRI signal power spectra were not corrupted by the presence of the mTMS equipment, we noticed a 122-Hz shift in the resonance frequency, additional peaks at ±31 kHz, and a stripe pattern and spatial shift in the fMRI images when mTMS coils were inside the bore and the capacitors were charged. To minimize the contribution of these effects on fMRI experiments, such as inducing false positives, either the capacitors can be maintained at a charged state (except during mTMS stimulation), or the charging of capacitors and mTMS stimuli are performed fully interleaved with the fMRI. Adjusting the stimulus parameters without frequent capacitor recharges can be achieved with pulse-width-modulation techniques, which could make the spatial shift deterministic and mitigate the charge-level dependent effects (Memarian Sorkhabi et al., 2022;Sinisalo et al., 2025). Furthermore, novel fMRI approaches with no acquisition delay (TE ≈ 0) and markedly better tolerance of frequency shifts could be used instead of EPI (Lehto et al., 2017;Paasonen et al., 2022).

Only a 6-ms delay was required after the mTMS pulse to have a negligible effect on the FID data acquisition with intensities up to 25% MSO. Based on logarithmic extrapolation, the required delays are within 10–20 ms for higher voltages (e.g., 67% MSO). Regarding fMRI acquisition during mTMS pulses, a slightly longer delay of ~40 ms was required to obtain artifact-free images with a GE-EPI sequence, commonly used in preclinical measurements. These results indicate that with the current setup, a 40–50 ms delay between the mTMS pulse and MRI volume acquisition would be enough for interleaved mTMS–fMRI. If considering a preclinical GE–EPI fMRI sequence with a 2000-ms repetition time, 15 slices with even distribution across the repetition time, and TE of 15 ms, slices are acquired with a 133-ms interval, and acquisition of one slice takes less than 40 ms. Therefore, a window of 90–100 ms exists between each slice where mTMS pulses can be applied, which is sufficient to prevent interference between the modalities when a delay of 40–50 ms would be needed. Another approach would be to squeeze the imaging slices to a time window of 600–700 ms, leaving about 1200 ms for the mTMS application every 2 s. A larger time window would also allow capacitor recharge. Notably, the required delay reported here (40–50 ms) is shorter or similar to those reported previously (Bestmann et al., 2003;Navarro de Lara et al., 2017).

The SPL increased logarithmically with increased stimulation intensity, and there were small differences depending on the stimulation orientations. At a 1-m distance from the coil, the acoustic noise levels were about 20–23 dB lower than at a 4-cm distance, so proper safety measures must be in place to prevent accidental mTMS pulses when personnel are working close to the bore, for example, during the preparation of the animal for the experiment. The safety limit for the peak SPL for humans (140 dB(C), computed with C-weighting) was exceeded during stimulation both outside and inside the MRI bore (Nyrhinen et al., 2024). Still, it remained at safe levels in the operation room. In general, laboratory animals exposed to continuous sound suffer from the same reactions to noise, ranging from auditory effects like temporary or permanent threshold shifts and tinnitus to non-auditory effects emerging from noise-induced stress, such as elevated heart rate and sleeping problems (Lauer et al., 2012;Turner et al., 2007). Experiments with mTMS in an ultra-high-field MRI should be performed with anesthetized animals, as in this study, carefully considering the animal safety regarding exposure to impulse sounds. Future development benefits from considering different approaches to reduce the SPLs, for instance, by using more efficient pulse waveforms to reduce the peak currents (Wang et al., 2023), incorporating double-containment coil mounting to reduce the mechanical coupling between windings and the outer case (L. M. Koponen et al., 2021), or designing coil windings optimized for minimum Lorentz forces to reduce wire movements and vibrations (Vílchez Membrilla et al., 2023).

As a proof of concept of the electronic control of stimulus orientation, we demonstrated that only specific E-field orientations and coil array locations generated MEPs. The measured MEP latencies (approximately 10 ms) and the presence of bilateral MEPs at a suprathreshold stimulation intensity are akin to those reported in previous studies (Boonzaier et al., 2020;Nieminen, Pospelov, et al., 2022;Rotenberg et al., 2010). Rats have primary and secondary motor cortices representing distinct levels of laterality in movement control and with different compositions of pyramidal cells, including axons projected bilaterally (Soma et al., 2017). Notably, mice exhibit bilateral forelimb movement representations in large cortical areas of a brain hemisphere, mapped with photostimulation applied in a 300-µm-spaced grid (Khanal et al., 2024). Therefore, the TMS-induced E-field (perpendicular focality 10–12-mm range; adult rat brain width is 15–20-mm range) likely depolarized neuronal pools from motor cortical representations with contralateral and bilateral projections even when the coils are placed a few millimeters apart. It is also possible that both coil array locations stimulated the same cortical region due to the widespread electric field patterns on the rat’s cortical surface (L. M. Koponen et al., 2020).

The highest amplitudes were obtained with stimuli delivered at 90°, −45°, 90°, and 135°, with predominant MEPs recorded from the left paw even when the coil array was placed over the right hemisphere (seeFig. 9A–B). The prevalence of MEPs from the left paw may be due to a higher motor threshold in the left hemisphere, as reported previously (Boonzaier et al., 2020), since stimulation intensity was based solely on the right hemisphere’s threshold. The high orientation specificity could be due to a localized motor cortical representation by neuronal ensembles aligned in laminar and columnar structures (Kätzel et al., 2011), as extensively studied in humans (Fox et al., 2004;Souza et al., 2018,^2022^;Weise et al., 2023), and further supported by the findings from stimulation of subcortical neuronal pathways with deep brain stimulation electrodes (Lehto et al., 2020;Slopsema et al., 2018).

MEPs were not measured inside the MRI scanner, but the observed MEP orientation sensitivity is not expected to differ from measurements outside the scanner. However, it should be noted that inside the scanner, TMS pulses might have slightly weaker effects due to dynamic interactions between the TMS and MRI coils (Peterchev, 2014). In addition, interleaved mTMS–fMRI on the rats were not recorded due to unexpected technical issues with the RF coil and the MRI scanner that succeeded the data recordings presented in this study. These recordings will be reported in a follow-up study.

The mTMS system described in this study can be directly translated for use in a traditional 3-T human MRI scanner. The only required modification is to design an mTMS coil set tailored to stimulate the human brain and compatible with RF coils for humans (Navarro de Lara et al., 2017,^2023^;Nummenmaa et al., 2015). Considering the lower MRI static field compared to the preclinical scanner and lower intensities for cortical stimulation, human TMS coils are exposed to much lower mechanical stress (M. A. Koponen et al., 2024) and are expected to be more durable than those for rodents. For 7-T MRI human applications, mTMS coils may still be exposed to lower mechanical stress. However, experiments might be impractical considering that extremely high acoustic noise from the mTMS pulse may pose health risks to the subject—extensive coil developments are needed to obtain safe, quiet, and efficient MRI-compatible stimulators.

## 5 Conclusion

Our system enables concurrent non-invasive neuroimaging and brain stimulation applications in a preclinical setting. This setup can be utilized to create and test a wide range of protocols to study the whole-brain network effects of neuromodulation (Bergmann et al., 2021;Mengotti et al., 2022;Tik et al., 2023;Yang et al., 2021) and treatments for neurological disorders (Dawson et al., 2018;Seewoo et al., 2018;Siddiqi et al., 2022).

## Supplementary Material

Supplementary Material

Supplementary Video

## Data Availability

The main data and code supporting this study’s results are available at OSF (https://osf.io/2qmzj/), in GitHub (https://github.com/vhosouza/mtms-mri-manuscript), and within the paper. Additional data and code can be requested from the corresponding author.
